# The Assessment of Esophagogastroduodenoscopy Tolerance a Prospective Study of 300 Cases

**DOI:** 10.1155/DTE.7.141

**Published:** 2001

**Authors:** Ashkan Farhadi, Jeremy Z. Fields, Seyed Hamid Bozorgnia Hoseini

**Affiliations:** 1 Department of Gastroenterology Mazandaran University of Medical Sciences Sari Iran; 2 Department of Internal Medicine Division of Digestive Diseases Rush Presbyterian St Lukes Medical Center Chicago IL USA; 3 General Practitioner National Iranian Oil Company (NIOC) Lavan Iran; 4 1725 W. Harrison St. Professional Building Suit 206 Chicago IL 60612 USA

## Abstract

*Background*: Esophagogastroduodenoscopy (EGD) is useful in the diagnosis and evaluation of dyspepsia. We investigated factors that might be associated with self-reported patient tolerance of EGD and therefore might serve as objective, reliable and useful surrogates for self-reported patient tolerance. We also investigated factors that might influence EGD tolerance.

*Study*: We prospectively evaluated 300 cases prior, during and after an EGD procedure. None received sedation.

*Results*: Seventy-nine percent of patients reported “good” tolerance of their EGD procedure. Other variables including (1) ease of intubation, (2) number and severity of retching episodes and (3) patient's cooperation during the endoscopic procedure, associated positively and robustly with patient self-reports of EGD tolerance. Evaluating the parameters that might predict EGD tolerance, only (4) age and (5) patient's gagging during Lidocaine throat spraying correlated closely with patient perception of EGD intolerance. Self-reported EGD tolerance did not correlate with gender, education level, body habitus (obesity), prior EGD experience, fear or anxiety about the procedure, procedure type or procedure duration.

*Conclusions*: Several parameters might be used instead of or in addition to patient perception of EGD tolerance. Age and patient gagging during Lidocaine throat spraying, but not patient fear and anxiety about the procedure can be used to predict EGD intolerance and used for selection of patients for sedation.


*Diagnostic and Therapeutic Endoscopy*, Vol. 7, pp. 141–147
Reprints available directly from the publisher
Photocopying permitted by license only

© 2001 OPA (Overseas Publishers Association) N.V.
Published by license under
the Harwood Academic Publishers imprint,
part of Gordon and Breach Publishing
a member of the Taylor & Francis Group.
All rights reserved.

### The Assessment of Esophagogastroduodenoscopy Tolerance a Prospective Study of 300 Cases

ASHKAN FARHADI[a]*, JEREMY Z. FIELDS[b] and SEYED HAMID BOZORGNIA HOSEINI[c]

[a]*Department of Gastroenterology, Mazandaran University of Medical Sciences, Sari, Iran;* [b]*Department of Internal Medicine, Division of Digestive Diseases, Rush Presbyterian St Lukes Medical Center, Chicago, IL, USA;* [c]*General Practitioner, National Iranian Oil Company (NIOC), Lavan, Iran*

*(Received 31 August 2001; In final form 31 August 2001)*

*Background*: Esophagogastroduodenoscopy (EGD) is useful in the diagnosis and evaluation of dyspepsia. We investigated factors that might be associated with self-reported patient tolerance of EGD and therefore might serve as objective, reliable and useful surrogates for self-reported patient tolerance. We also investigated factors that might influence EGD tolerance.

*Study*: We prospectively evaluated 300 cases prior, during and after an EGD procedure. None received sedation.

*Results*: Seventy-nine percent of patients reported "good" tolerance of their EGD procedure. Other variables including (1) ease of intubation, (2) number and severity of retching episodes and (3) patient's cooperation during the endoscopic procedure, associated positively and robustly with patient self-reports of EGD tolerance. Evaluating the parameters that might predict EGD tolerance, only (4) age and (5) patient's gagging during Lidocaine throat spraying correlated closely with patient perception of EGD intolerance. Self-reported EGD tolerance did not correlate with gender, education level, body habitus (obesity), prior EGD experience, fear or anxiety about the procedure, procedure type or procedure duration.

*Conclusions*: Several parameters might be used instead of or in addition to patient perception of EGD tolerance. Age and patient gagging during Lidocaine throat spraying, but not patient fear and anxiety about the procedure can be used to predict EGD intolerance and used for selection of patients for sedation.

*Keywords*: Esophagogastroduodenoscopy; Gastroenterologist; Patient selection; Tolerance

### INTRODUCTION

Esophagogastroduodenoscopy (EGD) is one of the most useful diagnostic procedures in the evaluation of dyspepsia and the patient's preference and tolerance is a major determinant in the physician's decision to use this tool as the first diagnostic procedure [1]. EGD tolerance is an important factor in determining patient

*Corresponding author. Address: 1725 W. Harrison St., Professional Building, Suit 206, Chicago, IL 60612, USA. Tel.: +312-942-8924. Fax: +312-666-5114. E-mail: ashkan farhadi@rush.edu preference, especially when this diagnostic tool is going to be used for patient follow-up. There are several methods to assess this tolerance, such as direct questioning of the patient [2–5], assessing the patient's cooperation during the procedure [3,6], counting the number of retching and gagging incidents per minute [3,6], determining oxygen desaturation determined by pulse oximetery [4,6,7] or measuring of serum cortisol level prior to and after the procedure [4]. There are several methods available to make EGD easier [2–4,6–10], including use of a Lidocaine-containing throat spray [2,3,8,11] or administration of sedatives orally [9], intravenously [4,6,10] or nasally [7].

This study was performed to assess possible correlations between factors that determine and predict EGD tolerance in non-sedated patients. The latter factors may be useful in predicting EGD tolerance prior to the procedure. Identifying those patients with poor tolerance prior to performing EGD, would be an ideal means to select those patients who would benefit most from sedatives.

### MATERIAL AND METHODS

This study was done on 300 consecutive cases that were referred to the Mazandaran University Endoscopic ward, From September 1998 to March 1999. These patients were all adults who had been referred to one of us (AF) for EGD. The endoscopist was a trained gastroenterologist that had performed more than 4000 EGD procedures during 6 years of practice in the same department. The instrument used was an Olympus Q-20 Gastroscope, a typical adult size endoscope. The endoscopic room and endoscopic nurse were the same for all of the patients. The endoscopic procedures included diagnostic EGD (both urgent and elective) and the interventions were biopsy taking and injection for upper GI bleeding. No cases with therapeutic intervention such as foreign body retrieval and stenotic dilatation were included in this study. Passing into the doudenum's second portion and retroflexion for examination of the cardia was done as part of the diagnostic EGD. None of these cases received any type of sedation prior to the procedure and the 44 cases who had received sedation only for medical purposes (e.g. comorbid heart disease and mentally incompetent cases) or per patient request were excluded from the study. It should be noted that using intravenous sedation is not a routine procedure in Iran where the study was performed.

After interviewing the patient and verifying the necessity for upper endoscopy, a questionnaire for each patient was completed by a nurse. Data were obtained for age, sex, level of education, smoking habit, patient's general condition, presence of obesity as determined by body mass index (BMI $\geq$ 29), history of previous EGD experience, and tolerance during the prior EGD procedure. The patient's pulse rate and pupillary size were determined and the patient was scheduled for EGD a few days after the first visit. The data regarding the patient's anxiety was not obtained in a few cases who needed emergency endoscopy. This was chiefly due to measurement difficulty and/or a pre-existing anxiety and tachycardia that they had due to severe bleeding or pain. In the endoscopy room all patients provided written informed consent and the questionnaire was completed for them. The presence of fear and anxiety about the procedure was evaluated by direct questioning of the patient, presence of pupillary dilatation (more than 2 mm greater than baseline) and measuring pulse rate (more than 20 per min greater than baseline, or more than 100 per min) just prior to the procedure. The data of pupillary size and pulse rate was disregarded in statistical analysis in those cases who had received any medication that affect the pulse rate and/or pupillary size.

After explaining the procedure to the patient and mentioning the necessity of his/her cooperation, the throat was sprayed while the patient was in a sitting position with five puffs of a 10% Lidocaine solution. Patient experiences of gagging during Lidocaine spraying were recorded. The patient was then put in left lateral decubitous position and the EGD procedure started with pharyngeal intubation. The intubation was recorded as good if the patient had no or little gagging and the endoscope passed easily from the throat with a little swallowing effort. Intubations were recorded as poor if there was severe gagging or difficult intubation despite the patient's swallowing efforts.

On completion of the intubation procedure, which was typically associated with the patient's relative relief, the Questionnaire administrator recorded the number and the severity of gagging and retching episodes. Retching that did not interrupt the patient's respiration and was not loud was regarded as mild. Retching, which interrupted the patient's rhythm of respiration or was loud was regarded as severe.

The endoscopist recorded the indication of and type of the procedure (diagnostic with or without biopsy taking and injection for upper GI bleeding), endoscopic diagnosis and the patient's cooperation during the procedure. The patient's cooperation in this case represented the endoscopist's point of view about EGD tolerance. Cooperation was regarded as good if, during the procedure, the patient made no or little movement that disturbed the endoscopist. In those with irritability or excessive movement that disturbed the physician, cooperation was recorded as poor.

The last question, that concerning the patient's opinion about the easiness of the endoscopic procedure, which was obtained just after the procedure, indicates the patient's perception of EGD tolerance. Because the patient's response to this question might not be straightforward, other measures were also used to assess patient's perception of EGD tolerance and to judge the easiness of the procedure. One such question was asking whether the patient preferred endoscopy as a follow-up diagnostic tool. The procedure was considered easy and the patient's perception of EGD tolerance was considered good, if a patient recalled the procedure as being easy or mildly difficult and still accepted upper endoscopy as the follow-up diagnostic tool. However if the patient recalled the procedure as being moderately to severely difficult or did not accept it as the follow-up diagnostic tool, the procedure was considered difficult or the patient's perception of EGD tolerance was considered poor.

The data obtained from these questionnaires were analyzed by SPSS computer software. The qualitative parameters were analyzed (1) by $2 \times 2$ tables, using the Chi Square test and (2) by calculating an Odds ratios. Student's *t*-test was used for parametric analysis of other variables such as age and number of retching incidents.

### RESULTS

The ages of the 300 patients enrolled in the study ranged from 18 to 87 years, with the mean being 40-years-old. Fifty-two percent were male and 48% were female.

As one objective, we tried to compare parameters that indicated EGD tolerance. These parameters directly or indirectly referred to the easiness of the EGD procedure and included (1) patient perception of EGD tolerance, (2) ease of intubation, (3) number, and (4) severity of retching episodes and (5) physician's perception of patient cooperation during the procedure.

Comparing the most important aspect of EGD tolerance, i.e. patient's perception of EGD tolerance, with other parameters that imply EGD tolerance, we found that the patient's perception of EGD tolerance correlated well with the endoscopist's assessment of patient cooperation during the procedure (Table I). Of patients perceiving good tolerance, most (83%) also exhibited good cooperation while of those patients perceiving poor tolerance almost 3-fold fewer (29%) had good cooperation during the procedure ($P < 0.0001$).

The patient's perception of EGD tolerance also correlated well with the ease of intubation (Table II). Of patients perceiving good tolerance, 75% had good intubation while only 34% of those with poor tolerance had good intubation ($P < 0.00001$). The other factors that correlated with patient perception of EGD tolerance were number and severity of retching episodes. Those with good tolerance had a mean of $3 \pm 0.05$ retching episodes per minute while those with poor tolerance had $5 \pm 0.06$. The difference between means was statistically significant ($P < 0.0001$). Also, 72% of patients with good tolerance

TABLE I Comparing patient self report EGD tolerance with physician assessment of EGD tolerance

| Patient's view/ Physician's view | Good cooperation | Poor cooperation | Total |
|---|---|---|---|
| Good tolerance | 196 (83%) | 40 (17%) | 236 (78.7%) |
| Poor tolerance | 19 (29%) | 45 (71%) | 64 (21.3%) |
| Total | 215 (72.7%) | 85 (28.3%) | 300 |

Chi-squares = 70.42; $P$ − value < 0.0001; Odd ratio = 12.3 (Confidence limit 95% = 5.98–24.45).

but only 25% of those with poor tolerance had mild retching ($P < 0.0001$).

We then evaluated parameters thought to influence EGD tolerance including age and gender. Gender was not associated with EGD tolerance, but age was. The patient's perception of EGD tolerance was significantly lower in those below 25 years of age compared to older adults ($P = 0.01$). At the same time scores for ease of intubation and patient cooperation were considerably greater in persons over 60 years compared to younger adults ($P = 0.04$ and $P = 0.005$, respectively).

The influence of education level was also evaluated. Patients were divided into several groups. Illiterates and those with only primary education were compared with those with high school education and/or higher education. There was no statistically significant difference in patient's tolerance, patient's cooperation and ease of intubation among any of the educational groups. Similarly, smoking, general health, urgency of the procedure and obesity did not correlate with EGD tolerance.

Thirty-six percent of the cases had a prior experience of EGD in their past medical histories. Of these cases, 76% recalled a good to fair tolerance in a previous EGD procedure. Neither the presence of a previous EGD experience nor the patient's tolerance during that procedure correlated with measures of EGD tolerance, in the present study.

Several parameters directly or indirectly indicated the magnitude of the patient's anxiety and fear about the endoscopic procedure. These were patient's answers to direct questioning about presence of fear and anxiety about the endoscopic procedure, presence of pupillary dilatation and presence of tachycardia. Forty-nine percent of cases had no or mild anxiety and fear while 51% had moderate or severe anxiety and fear about the procedure, when they were directly asked, prior to the EGD. Seventy-nine percent of the first group and 77% of the second group had good tolerance during the procedure and the difference was not statistically significant. Neither the presence of pupillary dilatation nor tachycardia was associated with different EGD tolerance during the procedure.

Only 10% of the cases had retching or gagging during throat Lidocaine spraying; 90% did not. In the latter group, 88% had good patient's perception of EGD tolerance, while only 55% of the former group had good tolerance ($P = 0.002$). Of the latter group, 70% had good intubation, while only 33% of the former had good intubation ($P < 0.0001$). Of the latter group, 75% had good patient cooperation during the procedure, while only 41% of the former group had good cooperation ($P = 0.0001$). In addition, patients in the latter group had a mean of $2.5 \pm 0.04$ retchings per minute, while those in the former group had a mean of $5.5 \pm 0.06$ during the EGD procedure ($P < 0.0001$).

The mean duration of the EGD procedure was $3.2 \pm 0.04$ min in patients with good EGD tolerance (patient's point of view) and $3.4 \pm 0.06$ min in those with poor tolerance. This difference was not

TABLE II Comparing patient perception of EGD tolerance with ease of intubation

| Intubation easiness/ Patient's view | Easy | Difficult | Total |
|---|---|---|---|
| Good tolerance | 178 (75.4%) | 58 (24.6%) | 236 (78.7%) |
| Poor tolerance | 24 (34.4%) | 42 (65.6%) | 64 (21.3%) |
| Total | 200 (66.7%) | 100 (33.3%) | 300 |

Chi-squares = 36.36; $P$ − value < 0.0001; Odd ratio = 5.69 (Confidence limit 95% = 2.99–10.89).

statistically significant. Similarly there was no difference between those with or without biopsy taking during the procedures with regard to patient perception of EGD tolerance. After the procedure, endoscopic diagnosis was established (e.g. non-ulcer dyspepsia, duodenal or gastric ulcer, gastric cancer). There was no significant difference in patient's perception of EGD tolerance among these diagnostic categories.

### DISCUSSION

Patient perception of EGD tolerance appears to be the most important determinant in patient acceptance of EGD as a diagnostic tool. However, several other parameters also indicate EGD tolerance including (1) ease of intubation, (2) number and severity of retching episodes, and (3) the endoscopist's assessment of the patient's cooperation during the EGD procedure. Thus, regarding our first research question—What factors determine EGD tolerance?—our findings indicate that these three variables can be used interchangeably or in parallel with the patient's perception of EGD tolerance. Walmslaey *et al.* showed a fair agreement between the assessment of overall acceptability of both endoscopists and patients in a study of 84 cases [12] while Thompson *et al.* found a poor correlation of these assessments in a series of 69 cases [13].

The second question—What factors influence EGD tolerance?—was asked because it could also help physicians decide which patients should be recommended for sedation during EGD. Although many physicians, prefer routine use of sedation during upper endoscopy, arguments persist as to whether anesthesia (via throat spray) or sedation is preferable for the patients [2,8]. Indeed, the usefulness of throat anesthesia or sedation in patients who need upper endoscopy is controversial [3,5,8,11,14–16]. It is generally agreed, however, that sufficient monitoring devices should be used during the endoscopic procedure in any endoscopic unit, especially if the patient is going to be adequately sedated [17]. An endoscopist usually uses his/her own experience to select those patients who would benefit more from sedation especially when adequate monitoring devices are not available. Identifying and characterizing those parameters that can be used in the selection process would be very useful in making this judgment.

Despite the widespread use of upper endoscopy in the evaluation of patients with dyspepsia, the literature contains few studies that deal with EGD tolerance. Furthermore, only a few of those studies that concern EGD tolerance evaluated the simple parameters that are actually used frequently in decision making by endoscopists. Our study deals with these rather simple and subjective parameters because the majority of them are practical and can be applied to the daily practice of endoscopists.

Our findings suggest that age is important. Patients $>60$ years of age are more likely to tolerate EGD without sedation while patients $<25$ are more likely to need sedation. This finding is consistent with previous studies [11,18].

In contrast, other demographic variables appear not to correlate with patient perception of EGD tolerance or to predict tolerance levels including sex, education level, body habitus (obesity) or general health status. This is a different result than previous studies which reported connections between male gender and higher education level with better patient satisfaction with endoscopy [18,19]. In a study of 37 cases by Gelly *et al.*, there was a strong trend for smokers to be more intolerant to intubation than non-smokers [20]. Such trend was not seen in our case series.

It might seem logical to use a patient's recall of prior EGD experience as a guideline for prediction of patients tolerance in a current procedure. In a prospective study by Campo *et al.* 31% patients undergoing gastroscopy for the first time had poor tolerance while only 26% of those with prior EGD experience had poor tolerance. Also the odds ratio for poor tolerance in the current procedure was 4.92 (CI = 1.93–12.5) for those who had poor tolerance in prior examination [19]. Our data indicate that neither the presence of a previous EGD experience nor the patient's tolerance during that procedure is a reliable predictor of current EGD tolerance.

Use of patient levels of fear and anxiety about the endoscopic procedure also seem logical. Indeed, these are currently the cornerstone indications for the use of sedatives during the EGD procedure. Moreover, studies support the logic in this as pre-procedure anxiety and fear, detected by direct questioning of the patient was one of the best predictors of the patient's tolerance and cooperation during the endoscopic procedure [11,18,19]. Soma *et al.* found that high anxiety score could be a predictor of poor tolerance in first time examines in a series of 201 cases [21]. Nevertheless, in our study neither of these parameters (measured prior to the procedure) correlated with the patient's perception of EGD tolerance or with the patient's cooperation. The reason for this discrepancy is not evident but it seems that other factors (such as age, throat sensitivity) might be involved rather than anticipated stressful situations affecting the overall reaction of the patient during an upper endoscopic procedure. Another possible reason is cultural differences as our patients were seen in clinics in Iran.

The patient's reaction (e.g. gagging) during throat Lidocaine spraying before the procedure was a good indicator of lack of EGD tolerance during the procedure. Those with gagging during throat spraying had a greater chance of having poor patient's perception of EGD tolerance ($P = 0.002$), difficult intubation ($P < 0.0001$) and poor cooperation ($P = 0.0001$). Ladas *et al.* mentioned a finger-throat test as a reliable predictor of EGD tolerance in his study in 1984 [22]. He evaluated the patient's tolerance of a throat examination with an endoscopist's index finger before and after throat anesthesia and found a good predictive value for this test. Assessment of gag reflex during throat spraying might be a more comfortable substitute for the finger-throat test mentioned previously.

The type and duration of the procedure were found to be predictors of EGD tolerance in some reports [12,18]. However in our study the type of endoscopic procedure (i.e. diagnostic versus therapeutic), duration of the procedure, or taking biopsies during the procedure, were not related to any parameter associated with EGD tolerance. Furthermore, EGD tolerance was not significantly different among different types of endoscopic indications or endoscopic diagnostic categories.

In conclusion, ease of intubation, number and severity of retching episodes and patient's cooperation during the EGD procedure could be used interchangeably with patient's perception of EGD tolerance. Young age and gagging during throat spraying are strongly correlated with patient intolerance and should therefore be considered as a useful guideline for predicting EGD intolerance and recommending the patient for sedation prior to EGD. Subjective fear and anxiety about the procedure, which have previously been well accepted indicators for sedation and history of good tolerance in previous EGD experience, may not be good predictors of EGD tolerance.

### References

[1] Stevenson, G.W., Norman, G., Frost, R., *et al.*, (1991) "Barium meal or endoscopy? A prospective randomized study of patient preference and decision making", *Clin. Radiol.* **44**, 317–321.

[2] Leitch, D.G., Wicks, J., El-Beshir, O.A., *et al.*, (1993) "Topical anesthesia with 50 mg of Lidocaine spray facilitates upper gastrointestinal endoscopy", *Gastrointest. Endosc.* **39**, 384–387.

[3] Jameson, J.S., Kapadia, S.A., Polson, R.J., *et al.*, (1992) "Is oropharyngeal anesthesia with topical Lidocaine useful in upper gastrointestinal endoscopy?", *Aliment Pharmocol. Ther.* **6**, 739–744.

[4] Ishido, S., Kinoshita, Y., Kitajima, N., *et al.*, (1992) "Fentanyl for sedation during upper gastrointestinal endoscopy", *Gastrointest. Endosc.* **38**, 689–692.

[5] Solomon, S.A., Kajla, V.K. and Banergee, A.K. (1994) "Can elderly tolerate endoscopy without sedation?", *J. R. Coll. Physicians London* **28**, 407–410.

[6] Chin, K.W., Tan, P.K. and Chin, M.K. (1994) "Sedation for gastroscopy: a comparison between Midazolam and Midazolam with Nalbuphine", *Ann. Acad. Med. Singapore* **23**, 330–332.

[7] Theissen, O., Boileau, S., Wahl, D., *et al.*, (1991) "Sedation with intranasal Midazolam for endoscopy of upper digestive tract", *Ann. Fr. Anesth. Reanim.* **10**, 450–455.

[8] Pereira, S., Hussaini, S.H., Hanson, P.J., *et al.*, (1994) "Endoscopy: throat spray or sedation?", *J. R. Coll. Physicians London* **28**, 411–414.

[9] Hedenbro, J.L., Ekelund, M., Aberg, T., *et al.*, (1991) "Oral sedation for diagnostic upper endoscopy", *Endoscopy* **23**, 8–10.

[10] Daneshmand, T.K., Bell, G.D. and Logan, R.F. (1991) "Sedation for upper gastrointestinal endoscopy: result of nationwide survey", *Gut* **32**, 12–15.

[11] Tan, C.C. and Freeman, J.G. (1996) "Throat spray for upper gastrointestinal endoscopy is quite acceptable to patients", *Endoscopy* **28**, 277–282.

[12] Walmsley, R.S. and Montgomery, S.M. (1998) "Factors affecting patient tolerance of upper gastrointestinal endoscopy", *J. Clin. Gastroenterol.* **26**, 253–255.
[13] Thompson, D.G., Lennard-Jones, J.E., Evans, S.J., *et al.*, (1980) "Patients appreciate premedication for endoscopy", *Lancet* **2**, 469–470.
[14] Davis, D.E., Jones, M.P. and Kubik, C.M. (1999) "Topical pharyngeal anesthesia does not improve upper gastrointestinal endoscopy in conscious sedated patients", *Am. J. Gastroenterol.* **94**, 1853–1856.
[15] Campo, R., Brullet, E., Montserrat, A., *et al.*, (1995) "Topical pharyngeal anesthesia improves tolerance of upper gastrointestinal endoscopy: a randomized double-blind study", *Endoscopy* **27**, 659–664.
[16] Fisher, N.C., Bailey, S. and Gibson, J.A. (1998) "A prospective, randomized controlled trial of sedation vs. no sedation in outpatient diagnostic upper gastrointestinal endoscopy", *Endoscopy* **30**, 21–24.
[17] American Society for Gastrointestinal Endoscopy (1995) "Sedation and monitoring of patients undergoing gastrointestinal endoscopic procedures", *Gastrointest. Endosc.* **42**, 626–629.
[18] Mahajan, R.J., Johnsoe, J.C. and Marshall, J.B. (1997) "Predictors of patient cooperation during gastrointestinal endoscopy", *J. Clin. Gastroenterol.* **24**, 220–223.
[19] Campo, R., Brullet, E., Montserrat, A., *et al.*, (1999) "Identification of factors that influence tolerance of upper gastrointestinal endoscopy", *Eur. J. Gastroenterol. Hepatol.* **11**, 201–204.
[20] Gelly, A.L., Farley, A., Boyer, J., *et al.*, (1998) "Influence of sex, age and smoking status on patient comfort during gastroscopy with pharyngeal anesthesia by a new benzocaine–tetracaine preparation", *Can. J. Gastroenterol.* **12**, 431–433.
[21] Soma, Y., Saito, H., Kishibe, T., *et al.*, (2001) "Evaluation of topical pharyngeal anesthesia for upper endoscopy including factors associated with patient tolerance", *Gastrointest. Endosc.* **53**, 14–18.
[22] Ladas, S.D. and Raptis, S.A. (1984) "Selection of patients for gastrointestinal endoscopy without sedation", *Gut* **25**, A550.

